# The Application of Microsatellite Markers as Molecular Tools for Studying Genomic Variability in Vertebrate Populations

**DOI:** 10.3390/cimb47060447

**Published:** 2025-06-11

**Authors:** Roman O. Kulibaba, Kornsorn Srikulnath, Worapong Singchat, Yuriy V. Liashenko, Darren K. Griffin, Michael N. Romanov

**Affiliations:** 1 Department of Animal Biology, National University of Life and Environmental Sciences of Ukraine, 03041 Kyiv, Ukraine; romankx37@gmail.com; 2 Animal Genomics and Bioresource Research Unit (AGB Research Unit), Faculty of Science, Kasetsart University, Chatuchak, Bangkok 10900, Thailand; kornsorn.s@ku.ac.th (K.S.); worapong.singc@ku.ac.th (W.S.); 3 Livestock Farming Institute, National Academy of Agrarian Sciences of Ukraine, 61026 Kharkiv, Ukraine; yurij2303@gmail.com; 4 School of Natural Sciences, University of Kent, Canterbury CT2 7NJ, Kent, UK; 5 Royal Veterinary College, University of London, London NW1 0TU, UK

**Keywords:** microsatellites, molecular markers, vertebrate species, animal populations, genetic diversity, alleles, genome functions, applications, PCR, marker-assisted breeding, conservation

## Abstract

Vertebrate molecular genetic research methods typically employ single genetic loci (monolocus markers) and those involving a variable number of loci (multilocus markers). The former often employ microsatellites that ensure accuracy in establishing inbreeding, tracking pan-generational dynamics of genetic parameters, assessing genetic purity, and facilitating genotype/phenotype correlations. They also enable the determination and identification of unique alleles by studying and managing marker-assisted breeding regimes to control the artificial selection of agriculturally important traits. Microsatellites consist of 2–6 nucleotides that repeat numerous times and are widely distributed throughout genomes. Their main advantages lie in their ease of use for PCR amplification, their known genome localization, and their incredible polymorphism (variability) levels. Robust lab-based molecular technologies are supplemented by high-quality statistics and bioinformatics and have been widely employed, especially in those instances when more costly, high throughput techniques are not available. Here, we consider that human and livestock microsatellite studies have been a “roadmap” for the genetics, breeding, and conservation of wildlife and rare animal breeds. In this context, we examine humans and other primates, cattle and other artiodactyls, chickens and other birds, carnivores (cats and dogs), elephants, reptiles, amphibians, and fish. Studies originally designed for mass animal production have thus been adapted to save less abundant species, highlighting the need for molecular scientists to consider where research may be applied in different disciplines.

## 1. Introduction

Molecular genetic research methods are currently at the forefront of investigations into animal genetics [1,2,3]. Predominantly, since its introduction in the 1980s, the polymerase chain reaction (PCR) has been the most routine tool used to solve many issues posed by selective breeding regimes [1,4,5,6,7]. Numerous methodological molecular approaches are now employed [2], but they basically break down into those that involve single genetic loci (monolocus markers) and those involving a variable number of loci (multilocus markers) [8]. Monolocus analysis can involve earlier biochemical (allozyme and protein) markers (e.g., [9,10,11,12,13,14,15]), microsatellites (e.g., [15,16]), single nucleotide polymorphisms (SNPs; e.g., [17,18,19,20]) and PCR–restriction fragment length polymorphism (PCR–RFLP; e.g., [21,22,23]), whereas multilocus studies can include random amplified polymorphic DNA (RAPD; e.g., [14,16,24,25,26]) and inter simple sequence repeat (ISSR) PCR-based analyses. A detailed exploration of research populations at the DNA level usually ensures a high degree of accuracy, enabling researchers to first determine differences between those populations and, thereafter, evolutionary changes when multiple populations are analyzed [27,28]. For instance, such investigations can be used to establish the level of inbreeding to trace the dynamics of the main genetic parameters across generations and to assess the genetic purity of a line [6,29]. Analyses of molecular markers lead to the very reliable identification of the species analyzed [30,31]; they allow populations or breeds of animals to be compared directly by genotype and thereafter ultimately correlated to the phenotype [31]. The use of monolocus and multilocus markers (either individually or in combination) in population studies enables the determination and identification of the spectrum of unique alleles (in molecular terms, DNA fragments) that are characteristic only to a particular population or breed [5,32,33,34,35].

In the study of species and populations thereof, all molecular genetic markers are useful, but microsatellites are especially so because of their high variability. A particular example is in studying and managing the marker-assisted breeding regimes of livestock [32,36,37,38]. Here, the application of microsatellites is a necessary element of the control over artificial selection to improve agriculturally important traits such as meat productivity, fertility, and disease resistance [39]. A highly relevant and early use of microsatellites was as primary molecular markers for developing genetic linkage maps in various plant and animal species (e.g., [40,41,42,43,44]). In some cases, the analysis of nuclear microsatellite loci is effectively used in combination with and complemented by the examination of the maternally inherited mitochondrial DNA (mtDNA) sequence, nuclear genes, and other molecular markers (e.g., [45,46]).

While many molecular markers (most recently single nucleotide polymorphisms—SNPs) are used for studying genomic variability in vertebrate populations, the aim of this article is to review the unique contribution and general utility of microsatellite analysis for genetic, ecological, and conservation purposes. Special emphasis has been given to comparative genomic strategies where information from well-described species such as human and farm animals is used to gain further insights into other species, especially those under threat. Due to space limitations, it would not be feasible to cover all of the numerous papers (currently, we estimate it to be near two million) of pertinent microsatellite research. Nonetheless, we offer several recent instances of these kinds of investigations to give insightful glimpses of microsatellite research in major vertebrate classes.

## 2. Microsatellite Markers: Their Features and PCR-Based Approach Used for Their Identification

### 2.1. What, Molecularly, Are Microsatellites and Why Are They Useful?

Microsatellites, otherwise known as simple sequence repeats (SSRs) or short tandem repeats (STRs), are usually monolocus and codominant [4,47]. They usually consist of 2 to 6 nucleotides in a specific sequence, repeating numerous times over, and are widely distributed throughout the genome of most animals and plants [40,48,49]. Accordingly, a 2-nucleotide repeat motif is referred to as a dinucleotide repeat (CA and CG repeats are the most cited [50]), with trinucleotide, tetranucleotide, pentanucleotide, and hexanucleotide repeats each denoting the length of the repeat unit. Microsatellites (SSRs or STRs) are noncoding, extremely variable in repeat length, and have an impact on how the genome functions [51,52]. They can be located both within the genes directly (i.e., introns, exons, etc.) and/or in noncoding regions of the genome [53,54,55]. Allele differences by a single repeat unit are typical. The main advantages of microsatellites as molecular research tools lie in

1. Their ease of use in PCR amplification strategies owing to specific DNA sequences that directly flank the motif itself. PCR primers can be derived and designed from these sequences and can then be strictly annotated for their uniqueness [56,57,58];
2. Their well-established localization in the genome and known proximity to other molecular markers through genetic mapping [41,43];
3. Their incredible level of polymorphism, which allows typing both different populations and single individuals with a high degree of probability [59].

**Figure 1:**
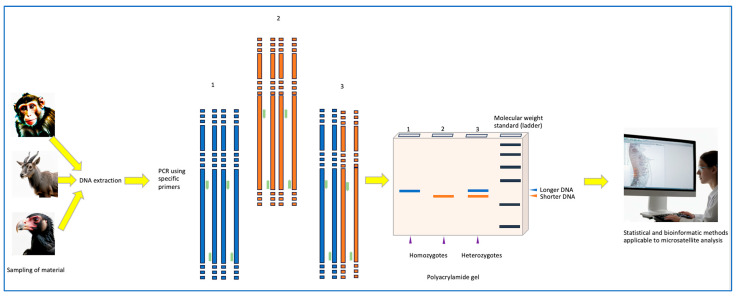
Schematic overview of microsatellite analysis, including its major steps such as sampling, DNA isolation, PCR amplification, gel visualization, and the use of computer-assisted statistical/bioinformatic applications.

Microsatellite analysis is, in essence, genomic fingerprinting, inherently identifying individuals, breeds, strains, and populations as required [40]. The specific owner (or owners) of a particular microsatellite motif can thus be determined, contributing to the effective determination of its origin [60,61,62,63]. A flowchart of microsatellite-based analysis is summarized in Figure 1.

### 2.2. PCR Detection of Microsatellites

In practical terms, PCR amplification of microsatellite loci (Figure 1) can be easily performed using an appropriate thermal cycler program [64,65]. Standard PCR reagents are used, including primers, dNTPs, a DNA polymerase, Mg^2+^, and a buffer; concentrations are optimized per target locus. For post-PCR microsatellite analyses (Figure 1), polyacrylamide gel (PAG) electrophoresis was originally widely used, especially in countries with poorly supported research labs. Most often, PAG electrophoresis is carried out under denaturing conditions. This is due to the need to determine precise differences in literally two nucleotides between different alleles, for which high-resolution systems are used. However, in some cases (especially if there is no or a limited access to sophisticated lab equipment), agarose gel electrophoresis can also be used, which, from the current point of view, is not always justified and efficient, since it can lead to an incorrect interpretation of the results due to the inability to distinguish between alleles (amplicons) [38].

Starting from the late 1990s, more advanced studies on the allelic polymorphism determination of microsatellite loci among biological species were carried out on appropriate high-throughput equipment [22,66,67]. The latter include DNA analyzers (sequencers) that allow us to unify an incoherent analysis of amplified fragments and, thereby, minimize the influence of the human factor on the decision-making process regarding the number and size of microsatellite alleles [38]. Currently, in addition to sequencers, other cutting-edge technologies are frequently employed, such as fluorescently labeled primers in multiplexes of microsatellite loci and other third-generation molecular approaches.

### 2.3. Genetic Diversity, Mutation Rates, and Heterozygosity: Microsatellites vs. SNPs

Microsatellites have been widely employed in genetic studies of animals and continue to be suitable codominant molecular markers, especially in those instances where more costly and high-throughput techniques, e.g., SNP array-based genotyping, are not available in some parts of the world [38]. In a comparative context, there is, in general, a correlation between the measures of genetic diversity using SNPs and microsatellites [68]. Genetic measurements from microsatellites and SNPs may, however, differ due to differences in population sizes, mutation rates, and the number of loci. While a greater number of SNPs may deflate genetic diversity estimations, high mutation rates can inflate them in smaller microsatellite marker sets [69]. Expected heterozygosity (*H_e_*) values can also be impacted by small population sizes [69]. SNPs produce lower *H_e_* values, *F*_ST_-based inter-population distances, and ancestry coefficients than microsatellites, according to earlier research [68].

## 3. Statistical and Bioinformatic Methods Applicable to Microsatellite Analysis

### 3.1. Classical Statistics

Similar to classical population genetic studies, appropriate statistics are essential when conducting microsatellite-based analyses (e.g., [70,71]; Figure 1). In particular, the frequencies of genotypes for microsatellite loci can be determined by the use of specific formulae. The allele frequency of codominant polymorphic microsatellite loci can also be calculated using maximum likelihood formulae and the error in genotype frequencies estimated. The error in allele frequencies can be established, as can the probability of allele frequency indices and the confidence interval of their variation. Genetic equilibrium is established using the Hardy–Weinberg formula and the level of genetic equilibrium in the distribution of genotypes in a population determined by the *χ*^2^ criterion. The level of observed (actual) heterozygosity (*H_o_*), in percentage terms, can be evaluated followed by the level of expected heterozygosity (*H_e_*). Thereafter, the coefficients of Wright’s [71,72] statistics can be established. The degree of divergence between populations can be assessed by the value of the *F*_ST_ coefficient, where an *F*_ST_ value between 0.00 and 0.05 implies a weak divergence, between 0.06 and 0.15 a medium one, that between 0.16 and 0.25 a large one, and one of >0.25 a very pronounced level of divergence [72]. Genetic distances can be computed using Nei’s [73] formulae and genetic similarity (*I*) determined.

For codominant microsatellite markers, certain formulae are applicable for estimating one more measure of the marker polymorphism degree, i.e., the polymorphic information content (*PIC*) [21,74,75,76], and *PIC* also serves as an indicator of a marker’s value in linkage analysis [77,78]. For microsatellite panels, *PIC* can be taken into account as a quantifying measure of accuracy and efficiency [75,79], along with match probability [80] and within-population variability [81].

### 3.2. Bioinformatic Software

To analyze molecular data, including microsatellite marker data (Figure 1), the FAO Commission on Genetic Resources for Food and Agriculture [57] suggested a software toolbox that includes a number of useful computer programs. Some of them can be used in more general genetic population studies, like Arlequin [82], FSTAT [83], GenAlEx [84], GenePop [85], Genetix [86], MEGA [87,88,89], PAUP* [90], PHYLIP [91], and Cervus [92]. Phylogenetic trees can be constructed using various computer program packages, e.g., PHYLIP [91] and MEGA [87,88,89]. There are also microsatellite-specific programs, e.g., those listed in Table 1.

To optimize the microsatellite marker panels used in genetic diversity and population genetic studies, a *PIC*-assisted ant colony optimization (ACO) algorithm approach has been proposed [79]. ACO utilizes a well-known, population-based, bioinspired, and heuristic optimization technique for resolving combinatorial issues [97,98]. Based on ant colonies’ natural behaviors, the ACO algorithm seeks to identify the best course of action by taking a number of costs or limitations into account [99]. Since *PIC* alone is not always useful to choose molecular markers, an improved selection methodology for microsatellite marker panel refinement, i.e., the *PIC*–ACO selection scheme, was developed. This procedure made it easier to obtain an optimal and reasonably priced microsatellite marker panel for the study of population genetic datasets and genetic diversity. This strategy could significantly lower financial obstacles to conservation, breeding, and population genetic evaluations [79].

To explore the non-random distribution and lineage specificity of microsatellite repeat motifs on vertebrate autosomes and sex chromosomes, the new tool Microsat Navigator [100] that enables the rapid and accurate study of perfect microsatellites in DNA sequences was created. Using it, microsatellite repeat patterns can be found across all genomic sequences, as was demonstrated for 186 vertebrate species. It was established that the abundance, density (number of loci/Mbp), length, and GC bias of microsatellites are significantly positively correlated with particular lineages. The most common motif in vertebrate genomes is (AC)*_n_* that exhibits specific patterns in closely related species. Mammalian and avian sex chromosomes have longer microsatellites, whereas autosomes do not. While the sex chromosomes of non-fish vertebrates have the lowest GC content, those in bony and cartilaginous fishes are characterized by high-GC microsatellites (≥50% GC). Accordingly, GC-rich microsatellites may be restricted to distinct clades by comparable mutational processes and selective factors. In addition to offering candidate microsatellites for functional examinations across the vertebrate evolutionary range, these discoveries should make it easier to investigate the involvement of microsatellites in sex genome differentiation [100].

## 4. Human and Livestock Microsatellite Studies as a “Road Map” for the Genetics, Breeding, and Conservation of Wildlife and Rare Breeds

Modern genetic advancements in livestock breeding serve as the foundation for productive and successful efforts to produce competitive, high-quality foodstuffs [32,35]. DNA technology is a major component of such global breeding regimes that have fueled population growth and alleviated hunger [37,101]. At the other end of the spectrum, however, many breeding regimes are applied using similar technology in order to preserve species and maintain biodiversity. In this regard, microsatellites are particularly useful. Indeed, the scope of application of microsatellite markers is very wide, with many studies being conducted on both plants and animals (e.g., [22,102,103,104,105]). In order to establish microsatellite variability in endangered species, the “road maps” provided by studies of various species of farm animals have proved especially useful [106]. For instance, microsatellite marker studies used to solve a wide range of problems in poultry breeding [107,108] were ultimately applied more widely. Moreover, for nine important livestock species, panels of 30 selectively neutral microsatellite markers (i.e., non-encoding proteins with no selection effect) were proposed by the Food and Agriculture Organization of the United Nations (FAO) and the International Society of Animal Genetics (ISAG)–FAO Advisory Group on Animal Genetic Diversity [57]. These species include cattle, buffalos, sheep, goats, horses, donkeys, camelids, pigs, and chickens. Compared to other microsatellite panels, the numerous datasets from finished characterization studies that have used ISAG–FAO markers enable new data to be compared with more breeds, especially for cattle, sheep, and goats.

In addition to population genetic studies, various alleles of microsatellite loci associated with the manifestation of quantitative traits are being searched for [43,109,110]. Of particular interest in this context are investigations aimed at finding an associative relationship between various alleles of microsatellite loci and resistance to viral and other infectious diseases, such as resistance to Marek’s disease in poultry [41,111]. When considering quantitative trait loci (QTLs) relevant to performance or disease resistance, microsatellites can be involved indirectly within a certain linkage group [112]. Several applications originally developed in livestock, including marker-assisted selection and traceability, have now been adapted for wildlife conservation through microsatellite-based tools. The primary applications of microsatellites include assessing population genetic parameters, phylogenetic analysis, the genetic differentiation of populations, genetic control in the process of marker-assisted breeding work, and the identification/certification of various breeds and lines [32,38,107,113,114,115].

In genetic and breeding studies of both farmed and wild animals, the use of molecular genetic markers significantly expands the capabilities of genetic analysis, which, in turn, makes it possible to establish inter- and intra-breed (i.e., linear or population) variability of individual regions of the genome [32,35,38]. It allows us to study the features of the genetic structure of experimental populations and trace the dynamics of variability in a number of generations. Microsatellites, as a specific class of molecular genetic markers, are widely employed to solve a number of tasks related to the genetic support of breeding work, e.g., to resolve issues related to the certification of poultry breeds and lines, assess the purity of breeding experimental lines, determine the level of consolidation of created groups, and establish the degree of genetic differentiation of populations [32,35,38]. Due to the high level of polymorphism of microsatellites, which is reflected in a larger number of alleles per locus compared to classical biallelic systems, microsatellite analysis can be used as an effective tool for studying genetic variability population differentiation. It helps us monitor the reduction in population size, which thus allows us to successfully address the entire spectrum of these issues in domestic and wild species (e.g., [116,117,118]). Microsatellite studies are applicable to all multicellular eukaryotes, including plants, animals, and fungi. Here, however, we focus our attention on vertebrate animals, predominantly mammals, starting with humans, where well-funded medical studies [27,119,120] provide the basis for research into apes and monkeys [121,122], as well as other animals. Thereafter we consider cattle and how it has been used to inform studies of other artiodactyls, then chickens as a standalone species and as a model for other birds, and then carnivores (dogs and cats), elephants, reptiles, amphibians, and, finally, fish.

### 4.1. Humans and Other Primates

The human genome remains the most studied of all species for medical reasons [4,50,123] and thus attracts the most funds. Up to 3% of the human genome is made up of microsatellites [123]. Researchers have been able to amplify these sequences in a number of non-human primate species, such as apes, baboons, macaques, and certain platyrrhine monkeys, owing to the prior isolation of microsatellites from the human genome [66,121,122,124,125,126,127]. Microsatellites are thought to have weak conservation among monkey lineages because they frequently accrue substitutions, insertions, and/or deletions [128]. Humans and monkeys have similar sequence lengths (up to 176 bp) for many conserved microsatellites, including *AP74*, which was found in New World monkeys [27,129]. The majority of the telomeric region up to 15 kb on human chromosomes can be formed by repeating certain microsatellite monomers, and they are known as (TTAGGG)*_n_* sequences [120,130,131]. Some nucleoproteins, including telomeric repeat binding factor 1 (TRF1), telomeric repeat binding factor 2 (TRF2), and protection of telomeres 1 (POT1), can attach to these telomeric repeats to create a complex known as “shelterin” [132], which then interacts with a ribonucleoprotein [133]. This complex protects chromosomal ends from deterioration and aids in DNA repair mechanisms [134]. Primate telomeres include the microsatellites (CCCTAA)*_n_*, (CCCCAA)*_n_*, and (CCCTCA)*_n_* [119], while subtelomeres only contain (CCCGAA)*_n_* [135].

According to population-level research based on microsatellites [136], macaque genomes were found to be more nucleotide diverse than human genomes. In wild rhesus macaques, cross-species amplification of microsatellite loci has revealed decreased genetic differentiation as a result of inbreeding [137].

### 4.2. Cattle and Other Artiodactyla

Among domesticated artiodactyl species, the examination of beef and dairy cattle populations by the complex of microsatellite loci and the generation of the genetic structure data can be a valuable source of information in terms of both the preservation of the gene pool of breeds and the control of genetic processes in artificially reproduced animal populations [38,46,94]. The interest in cattle for both beef and dairy means that microsatellite investigations have been well-funded, though less so than in humans. Considering the potential value of the researched breeds as carriers of specific biological and economic features for specific geoclimatic breeding and exploitation conditions, it would be advisable to analyze changes in their genetic structure by microsatellite markers compared with the data of previous years of research or with the data of the initial stocks involved in the creation of a breed. As a result of these microsatellite-assisted studies, valuable information can be obtained regarding the examined breed or population, which can be correctly used for further analysis and the development of the appropriate breeding or conservation measures [32,38].

It would not be practicable here to cover the myriad of publications of relevant microsatellite research in cattle given the space constraints. We thus provide a selection of recent examples of such studies. For instance, using 30 microsatellite markers, Koul et al. [138] molecularly characterized a native short statured cow population of Nattukuttai (*Bos indicus*) in southern India. An average of 9.8 alleles per locus and a mean *PIC* value of 0.763 indicated a significant degree of genetic diversity and high marker polymorphism. A considerable number of loci showed deviation from the Hardy–Weinberg equilibrium, suggesting potential genetic factors like selection or population structure fluctuation. There was no recent significant population decline among the Nattukuttai according to bottleneck analysis. Exhibiting a unique genetic profile, Nattukuttai deviated from a common ancestor that also produced the Malai Madu and Kangayam cattle and demonstrated genetic differentiation from the other populations [138].

Ali et al. [46] explored the genetic variability of the Gabrali cattle, a multi-purpose native breed of great economic importance in Khyber Pakhtunkhwa, Pakistan, using 12 microsatellite loci. The breed was characterized by substantial genetic diversity, with an average of 8.8 alleles per locus; observed and expected heterozygosity of 0.58 and 0.50, respectively; a mean *PIC* of 0.59; and an *F*_IS_ (inbreeding coefficient) of 0.056. According to the Hardy–Weinberg equilibrium, the microsatellite study showed a normal allelic distribution throughout the breed. It was determined that the Gabrali cattle is genetically diverse and does not face the threats of inbreeding and genetic bottlenecks [46].

Ladyka et al. [139] examined the gene pool of a local population of endangered Lebedyn cattle compared to other Ukrainian breeds and populations. Ten of the FAO-ISAG-recommended microsatellite loci were used in the population genetic structure study. On average, five alleles per locus were found. Most of the loci under investigation were useful informative markers (*PIC* > 0.5). The Lebedyn population was in genetic equilibrium except for two loci. Although there was sufficient genetic variability, the average *F*_IS_ value showed a propensity for inbreeding (due to an increase in homozygous animals), indicating that fewer animals in the populations compared would have a negative impact on the genetic diversity of local cattle breeds. This mostly affected the Ukrainian Grey and Lebedyn breeds, while the Red Steppe breed, with a larger population size, and an imported Grey Bulgarian breed (one of the oldest aboriginal breeds in Bulgaria) were not at risk of inbreeding. It was concluded that microsatellites can be a suitable tool for molecular marker-assisted selection (MAS) and breeding in order to monitor the detrimental effects of artificial reproduction on the gene pool of small cow populations [38,139].

As examples of wild artiodactyl species for which microsatellite marker research is a primary tool for developing ex situ and in situ conservation management, the Korean goral (*Naemorhedus caudatus*), as well as the Chinese goral (*N. griseus*), can be considered [64,140,141]. Here, a core set of 11 microsatellite markers previously approved for cross-species amplification in five Caprinae species, including the Korean goral [64], is of particular applicability and efficiency. This set was also instrumental for population genetic studies in the Chinese goral that is native to Southeast Asia and has a vulnerable status due to overhunting [141]. Despite the absence of a bottleneck, the low level of genetic variation was probably caused by inbreeding. In captive programs, estimates of small effective population sizes and restricted founders, along with wild-born individuals within subpopulations, tend to reduce genetic variation over time. This results in low reproductive fitness and restricted ability to adapt to environmental change, hence increasing the danger of extinction. The management of captive populations as evolutionarily significant units with various genetic backgrounds offers an effective technique for population recovery in the Chinese goral [141]. Using the *PIC*–ACO algorithm approach, it was verified that a complete set of 11 markers was required for the most accurate population genetic assessment in this species [79].

In Southeast Asia, there is the mouse-deer, a primitive forest ungulate and the smallest ruminant [142]. Although the greater mouse-deer (*Tragulus napu*) is presently of least concern, human hunting and habitat fragmentation brought on by continuous deforestation are the main risks to this species. The usage of 11 microsatellite loci was helpful in the genetic monitoring of the last greater mouse-deer captive population on the Thai mainland prior to reintroduction. While there was not a historical bottleneck, a significantly reduced effective captive population size was noted, along with inbreeding patterns. The likelihood of a population decline and eventual extinction has increased due to these conditions that have decreased their reproductive fitness and capacity for environmental adaptation. Because of efficient animal care and reproduction, demographic analysis indicated a recent increase in the number of captive animals. According to these data, the primary determinant of allelic diversity (i.e., number of alleles) is population size at equilibrium [142].

### 4.3. Perissodactyla

The features of microsatellite variation in Perissodactyla have been studied using various horse (*Equus caballus*) breeds as an example [143]. For instance, using 11 SSRs, Shelyov et al. [143] investigated microsatellite variability in populations of three breeds: the Hutsul, Thoroughbred, and Ukrainian Saddle. The maximum polymorphism level was found in the native Ukrainian Saddle population, with an average number of 14.3 alleles per locus, 89% being breed-specific alleles, and nine loci containing breed-specific alleles. Thus, the minimum polymorphism level was noted for the Thoroughbred population. A high number of different genotypes were found for individual microsatellite loci in the Thoroughbred and Hutsul breeds. For the three breeds, high values of expected heterozygosity (0.707–0.865) were found, with positive *F*_IS_ values conforming to 3–8% of inbreeding, as well as a genetic consolidation of the studied populations.

Microsatellite analysis has been extensively employed in horse populations worldwide for various purposes, including parentage testing, population structure assessments, and genetic conservation. In Korea, one of the earlier efforts in microsatellite-based parentage verification was conducted by Cho and Cho [144], who used 16 microsatellite markers to establish an identification system for Korean native horses. Their study demonstrated the effectiveness of these markers in distinguishing individuals and identifying mismatched pedigrees, thereby laying the groundwork for later applications in genetic monitoring and the conservation of native horse populations. In Kazakhstan, a study on 435 horses from the Kushum and Mugalzhar breeds revealed high genetic variability, detecting 136 alleles across 11 STR loci with minimal population differentiation (*F*_ST_ < 0.05), indicating a shallow structure and significant gene flow between regional populations [145]. Commonly used microsatellite loci, such as *AHT4*, *AHT5*, *HTG4*, *HTG7*, and *HMS3*, showed high *PIC* (>0.5), supporting their usefulness in breeding monitoring and local breed conservation [145].

Sukri et al. [146] reported moderate genetic diversity in the Sumbawa horse, an endemic Indonesian breed, based on 24 individuals from two populations. The study found signs of inbreeding and population differentiation, highlighting the breed’s uniqueness and the importance of its conservation.

In donkeys (*Equus asinus*), microsatellite markers have also been effectively applied, though fewer studies exist compared to horses. A study in South Korea using 15 microsatellite loci across 79 donkeys and 100 horses (including Thoroughbreds and Jeju Halla horses) demonstrated moderate heterozygosity in donkeys (*H_e_* = 0.5635; *H_o_* = 0.4861) and clear genetic distinction from horses, underscoring the usefulness of these markers for donkey-specific parentage testing and conservation [147]. Complementing this, Park et al. [148] analyzed nearly 6000 Thoroughbred horses over a decade in Korea and confirmed the continued utility of microsatellite markers in parentage verification. Their study highlighted the consistency of genetic diversity levels over time and emphasized the robustness of the current panel of 15 STRs, including 12 recommended by ISAG. The latter remains a valuable tool for maintaining the genetic integrity of horse populations despite the increasing global shift toward SNP technologies.

Beyond their use in diversity assessments and parentage verification, microsatellites have proven especially valuable in conservation genetics. As reviewed by Wang et al. [149], microsatellites offer a robust tool for evaluating the genetic structure, detecting inbreeding, and informing breed-specific management, particularly in endangered donkey populations across Asia and Europe. Their species specificity, high polymorphism, and co-dominant inheritance make them uniquely suited for monitoring rare breeds such as the Chinese native donkeys, the Italian Pantesco, and the Mongolian wild ass, all of which face genetic erosion and require structured conservation frameworks.

### 4.4. Chickens

Like cattle, chickens attract considerable research funding into their molecular markers for meat and other products (in this case, eggs) [38]. Chickens are also a classic model for developmental biology [20,150,151,152]. There are many examples of using microsatellites to analyze chicken populations, both domestic and wild [15,60,62,153]. Among the most recent, a study on the population structure and genetic diversity within the Canarian population, as well as between the Canarian, Spanish local, and commercial populations [154], is worth of mentioning. The *F*_ST_ value in the Canarian population was comparatively high (0.179) relative to other commercial strains and local Spanish breeds (0.164–0.195). The neighborhood network analysis revealed that the Canarian varieties did not cluster with the other Spanish breeds, whereas there was the difference between the Rubilana variety and the four others of the Canary Islands, as confirmed by the STRUCTURE analysis [155]. When comparing the Canary Islands chicken population to other Spanish and commercial breeds, its genetic profile was distinct. Except for the Rubilana population that might be accepted as a genetically different variety, the hypothesis that the existence of genetic varieties in the Canary Islands is based on feather color was entirely rejected [154].

Aoki et al. [156] developed a microsatellite-based high-resolution melting (HRM) technique to successfully create a unique screening method for Nagoya breed discrimination. Using four Nagoya meat samples and twelve from other Japanese native and foreign broiler breeds, a primer set for HRM analysis was created to amplify the CA repeat in the *ABR0417* microsatellite marker. While the sequences of the twelve other chickens differed from those of the Nagoya breed, the sequences of the four Nagoya breed birds were identical to the breed’s *ABR0417* reference sequence. The Nagoya breed chickens had different melting curves and peak plots than other chickens. These findings show that the HRM-based approach is a straightforward genetic test that uses the *ABR0417* marker to identify the Nagoya breed [156], and similar microsatellite-based assays can be developed for breed identification purposes.

Many studies focus on the genetic variability and differentiation of various breeds and lines, for instance, amongst Ukrainian chickens of the layer and dual-purpose types [38,157,158,159,160]. These included the Plymouth Rock White, Birkivska Barvysta, Poltava Clay, and Rhode Island Red breeds and their strains. A subset of 14 microsatellite loci consisted of ten selectively neutral markers recommended by ISAG-FAO [56,57] and those associated with the manifestation of resistance to neoplastic diseases (*MCW0245*, *MCW0257*, *MCW0282*, and *MCW0288*). The mean number of alleles per locus was 4.7, whereas all loci were polymorphic. According to the *PIC* values, the total number of highly informative markers was ~45%. Significant divergence (*F*_ST_ = 0.19) was found between the examined populations. The largest genetic differences were found between the Plymouth Rock White and Rhode Island Red breeds (65.9% of differences), and the smallest ones were found between the Plymouth Rock White and Poltava Clay breeds (32.3%). In addition, the level of genetic differentiation of subpopulations of Ukrainian meat–egg chickens (G-1, G-2, G-3, G-4, and C), as well as Lines 02 and 38 of the Rhode Island Red breed, was established using eight microsatellite markers. It was found that the most genetically distant subpopulations were G-1 and G-4 (28.8% differences) compared to the closer subpopulations G-2 and G-3 (13.3%). Lines 02 and 38 of the Rhode Island Red breed demonstrated minimal differences (7.9%) [38,160]. These studies are critical for understanding the features of the genetic component of the breeding nucleus in poultry. Moreover, identifying the genetic structure of local chicken breeds is pivotal from the standpoint of the issue of preserving their gene pool to mitigate risks for the poultry industry that is focused on a few highly productive and selected breeds and lines [38].

A larger set of 28 ISAG-FAO-suggested [56,57] microsatellite loci has been implemented for evaluating gene pool diversity and differentiation patterns in indigenous chickens and red junglefowl (*Gallus gallus*) across Southeast Asia (e.g., [161]). For instance, a highly diverse Mae Hong Son chicken population has unique allelic gene pool patterns, i.e., a distinct DNA fingerprint in contrast to other breeds and red junglefowls. It has been suggested that the Mae Hong Son chicken originated as a crossbreed between Thai indigenous village chickens and red junglefowls, as evidenced by the discovery of genetic introgression of certain gene pool components from the domestic and wild birds [161]. A comparison of microsatellite genotypes in two indigenous breeds, Pradu Hang Dam and Samae Dam, showed their high genetic variability and a partial overlap of their gene pools, indicating that the Samae Dam may be a variety of the Pradu Hang Dam. One Samae Dam subpopulation shared a gene pool with the red junglefowl that partially overlapped [162]. In a genetic admixture and diversity study of Lao Pa Koi fighting cocks from Thailand [163], 182 alleles, on average 6.5 alleles per microsatellite locus, were found. The red junglefowl and Lao Pa Koi chickens shared a partial gene pool, according to the microsatellite data. Two other Thai fighting cock breeds, Lueng Hang Khao and Pradu Hang Dam, demonstrated hitchhiking selection at 28 microsatellite loci, suggesting directional selection in fighting cocks [164]. An analysis of the gene pool of Thai fighting chickens also showed admixture with other domestic breeds and a small effect from red junglefowl. The observed genetic structure within these breeds seems to be explained by selection for cockfighting and decorative features.

According to Budi et al. [62], the Chinese black-boned chicken gave rise to the Thai local chicken breeds Chee Fah and Fah Luang that also had introgression from red junglefowls and other chicken breeds. An investigation of population structure showed that the populations of the Chee Fah and Fah Luang chickens constituted a distinct cluster from those of other Thai domestic breeds and red junglefowls. During their domestication and population growth, these regional chicken breeds most likely developed special and beneficial characteristics, like ecological adaptability [62]. Rare Dong Tao chickens from Vietnam (also called Dragon Chickens because of their huge reddish feet) are a distinctive and productive poultry variety. Luu et al. [165] suggested that directional selection brought on by environmental adaption pressures contributed to this breed’s microsatellite-based genetic similarities to indigenous, local chicken and red junglefowl populations from Thailand.

Wild progenitor red junglefowls (*G. gallus*) from 12 populations, representing two subspecies (*G. g. gallus* and *G. g. spadiceus*), and chickens from ten native Thai breeds were the subjects of a comprehensive genetic study employing the same 28 microsatellite DNA markers [60]. High genetic diversity was found in the red junglefowl groups using Bayesian clustering analysis, as was also confirmed by Singchat et al. [153]. These findings indicate that there were sizable ancestral populations of native Thai chickens and that the domestication process did not encompass all of the gene pool of the red junglefowl population [60]. While the red junglefowl germplasm is widely dispersed throughout Thailand, further successful reintroduction of this wild species depends heavily on its gene pool [153]. For genetic diversity research in chicken and red junglefowl populations, the *PIC*–ACO selection strategy can also be used to choose the best microsatellite panel with varying accuracy loss tolerances [79].

### 4.5. Other Birds

The isolation and characterization of novel microsatellite loci is a pivotal approach for population genetic studies in other birds, including domesticated, captive, and wild species and strains. In particular, Bei et al. [166] reported 12 new markers for the rare and endangered Hume’s pheasant (*Syrmaticus humiae*). No linkage disequilibrium was discovered between locus pairings, and four loci displayed deviations from the Hardy–Weinberg equilibrium. These informative microsatellite markers have been a valuable molecular tool in subsequent research on the evolutionary history and population genetic makeup of *S. humiae*. In a study by Bei et al. [167], high genetic diversity in Hume’s pheasant was revealed. This bird may face genetic obstacles in two Chinese provinces because of rivers and a national route. Furthermore, despite population decreases in the previous century and the bottleneck that occurred about 5000 years ago, genetic distinctiveness has remained for Hume’s pheasants. In contrast, microsatellite locus analysis of a Thai captive-bred Hume’s pheasant flock [168] showed a weak population structure and much less genetic differentiation. There was no bottleneck despite the limited genetic variability; however 12 microsatellite loci were useful in indicating likely inbreeding. These discoveries offer a wealth of information to optimize genetic diversity and improve the efficacy of upcoming conservation initiatives for Hume’s pheasant populations in captivity and the wild.

To support the efficient conservation management of threatened Asian woolly-necked storks from three captive breeding programs (*Ciconia episcopus*), 13 microsatellite loci were used to examine the genetic diversity and population structure [169]. One population showed inbreeding and a very small effective population size, indicating that several generations kept in captivity had caused harmful genetic problems. In contrast, another population showed signs of a recent demographic bottleneck. The historical changes in the genetic diversity and demographics of endangered oriental storks (*C. boyciana*) from founder introduction, captive propagation, and reintroduction in species recovery were examined using microsatellite markers and demographic data [170]. It was discovered that prolonged captive propagation saturated and stabilized the level of genetic variation. These results imply that, in the early stages of reintroduction, balancing the genetic variety of captive and reintroduced storks could be achieved with regular assessments of genetic diversity and selection for releasing individuals using efficient genetic markers.

Studies by Romanov et al. [44,171] provided microsatellite variation data for the endangered California condor (*Gymnogyps californianus*), which can be used to select polymorphic markers and investigate a range of genetic factors and phenomena. They work well, for example, in kinship and paternity (parentage) analysis instances involving condor chicks that are now living in the wild. In particular, a parentage study of chicks produced from condor eggs placed in the wild was successfully conducted using a panel of these polymorphic microsatellite markers [172]. Additionally, it helped find a few instances of facultative parthenogenesis in California condors, which was the first time this asexual reproductive event in a bird species was detected using molecular markers [173]. Utilizing a collection of specially developed 123 anonymous microsatellite loci, 121 members of a reference condor population were genotyped. Fifteen linkage groups/subgroups were found as a result, creating a first-generation condor genetic linkage map [171].

### 4.6. Dogs

Dogs are part of popular culture because of their long-standing role as companion animals [174]; hence, microsatellite research is reasonably well funded. Along with various genetic analysis purposes, microsatellite length polymorphism markers can be used for canine parentage testing [175]. For example, 12 SSR markers that were carefully selected, validated, and approved were recently used to test the markers’ effectiveness in popular dog breeds kept in India (not necessarily native breeds): the Labrador, German Shepherd, Pug, Mudhol Hound, Tibetan Mastiff, Gaddi dog, Beagle, Belgian Malinois, Pointer, and Cane Corso. The effective number of alleles varied from 3.6 to 15.2, while the mean number of alleles per locus varied from 5 to 29. Over 0.73 was the expected heterozygosity. The breeds under study showed no signs of inbreeding, according to the population inbreeding coefficient (*F*_IS_), and the *PIC* values exceeded 0.65. These findings showed that the 12 molecular markers that were chosen are sufficient for establishing dog parentage [175].

The Bangkaew and Thai Ridgeback dog breeds, which originated in Thailand, are renowned for their distinctive characteristics [176,177]. The Bangkaew breed’s significant genetic variation and low risk of inbreeding were discovered. Identifying Bangkaew dogs was successful when utilizing a 15-loci microsatellite panel. Additionally, a refined 10-loci microsatellite genotyping panel offers enhanced identification testing efficiency, fostering cost and time effectiveness [176]. Similarly, the current population of Thai Ridgebacks has a high level of genetic diversity and little inbreeding. The flow of genetic material among Thai Ridgeback owners successfully conserved the genetic diversity; therefore there were no indications of bottlenecks [177].

The genotypic variability within and among 28 breeds that constitute the seven recognized breed groupings of the American Kennel Club was evaluated [174], building on prior studies of dog populations. Differentiation within breeds was investigated using 100 autosomal microsatellite markers spread throughout the canine genome. Breed-to-breed genetic relatedness was less clear-cut, while the autosomal microsatellites set proved helpful in characterizing genetic variation within breeds. It was also concluded that SNPs would probably be needed in order to determine breed phylogeny more precisely [174].

### 4.7. Cats

Like dogs, there is considerable interest in cats (and, by extension, their microsatellites) because of the pet industry [42,63]. Effective and reliable genetic data collection in domestic and wild cats has been made possible by recent developments in microsatellite genotyping; high-throughput sequencing methods, e.g., amplicon sequencing; and bioinformatics [178,179]. To cross-genotype Felidae species, a multiplex panel of 85 co-amplifying tetranucleotide microsatellite markers (Feliplex) was created. In support of their economical genetic research and conservation monitoring, Feliplex was confirmed on nine felid species from the genera *Felis*, *Panthera*, and *Prionailurus* that are found in India [179]. In the genic and genomic sequences, the frequency of microsatellites in domestic cats (*Felis catus*) was measured and compared to that of wild cats, such as the tiger (*Panthera tigris*) and cheetah (*Acinonyx jubatus*). Compared to wild cats, domestic cats have the highest frequency, relative abundance, and density of microsatellites. Over 40,200 primer pairs were composed using genic sequences to provide genetic resources to members of the Felidae family. The molecular genetics of a given cat’s identification and population structure may be ascertained with the use of these markers [178].

The well-known ancient Thai domestic breeds are the Siamese (Wichien Maat) and Korat cats. Using 30 microsatellite markers, their genetic diversity was examined by Ubolrat et al. [180]. Two Thai native cats had a moderate degree of genetic diversity, with a higher inbreeding coefficient than previously thought. In another study [63], a larger sampling of five Thai domestic cat breeds were surveyed for genetic diversity and structure. Based on 15 microsatellites, all breeds exhibited high genetic diversity (*H_o_* and *H_e_* > 0.5). Compared to the other breeds, the Siamese and Korat breeds displayed distinct gene pool patterns. Presumably, Thai cat breeds originated in isolated areas with similar racial origins have experienced allele fixation for unique morphological features [63]. Lipinski et al. [181] evaluated the modern evolution of cats, including their domestication, using microsatellite analysis. Information was gleaned from >1100 cats in 17 random-bred populations. They were sampled from a total of 22 breeds that derived from five continents. The studies confirmed previous reports of the Mediterranean region as the most likely place of original domestication. Genetic diversity has stayed broad in cats globally, but there are measurable genetic clusters around the Mediterranean basin, as well as Europe/America, Asia, and Africa. Evidence suggests that Asian cats diverged early and expanded in relative isolation, and the majority of breeds appear to be derived from indigenous cats corresponding to their purported regions of origin. There are exceptions however, with Japanese and Persian bobtail cats more aligned with the European/American cluster than those of the Mediterranean basin. Moreover, three recently emerged breeds were not distinct from their parental breeds of origin. Pure breeding by enthusiasts is, as expected, associated with genetic diversity loss, but this loss does not appear to correlate with age, nor the popularity of the breed.

As in many other species, a cat genetic linkage map was developed using autosomal and X-linked microsatellite loci in the feline genome [42]. The map has been a useful resource for mapping phenotypic diversity within the species and connecting it to gene maps of other animals, including humans.

### 4.8. Elephantidae

Polymorphic microsatellite markers have offered good opportunities to study both elephant and mammoth genetics. They have been isolated, characterized, and well analyzed in the African elephant (*Loxodonta africana*) [45,67,182,183]. In the Asian elephant (*Elephas maximus*), Fickel et al. [184] reported microsatellite examinations of individuals categorized by one of two matrilinear mtDNA haplotype clades (*α*_h_ and *β*_h_). The related nuclear microsatellite genotypes, coined as *α*_nuc_ and *β*_nuc_, showed a significant genotypic difference. In this physically homogeneous species, genealogically diverse variants are thought to be a sign of cryptic speciation. Interestingly, bulls were the cause of this differentiation, whereas the consideration of cows alone produced no differentiation. In three captive (domestic) Thai *E. maximus* populations with a wide range of population-specific gene pool estimates, Ariyaraphong et al. [30] established a high variation in genotypes from 18 microsatellites. However, the population’s genetic diversity decreased over the next 50 generations at all loci with restricted one-male polygyny mating, according to microsatellite data. By providing an assessment of the population status of captive elephants in a certain geographical area (such as Thailand), guidelines for their management can be implemented. Thereafter, the long-term preservation of captive elephant populations can be promoted based, in part, on enforced legislature. Studies such as [30] provide a road map for a more optimistic sustainable future in synergy with various sustainable development goals. A microsatellite-driven, evidence-based set of standards and guidelines, alongside enforceable regulations, for elephants held in captive breeding populations assists in this endeavor. More studies pertaining to the genetic variation and differentiation of a greater number of Asian elephants need to be performed and expanded to other areas. Collaboration with captive breeding initiatives, the government, and tourist agencies will also encourage the maintenance of healthy, sustainable populations of captive elephants in the places that are most needed [30].

In a paleogenetic study [59], woolly mammoths (*Mammuthus primigenius*) in northeastern Siberia were investigated for their demographic history from before 60,000 years ago to their eventual extinction around 4000 years ago using four autosomal microsatellite DNA markers. The end-Pleistocene reduction in mammoth autosomal genetic variation was revealed by this microsatellite genotyping.

### 4.9. Reptiles

The most current sophisticated genome-scale analysis of primarily microsatellites and some other satellites (with the repetitive portion of the genome termed the “repeatome”) has shown that they are remarkably abundant in squamate reptile genomes [28,185]. According to the amplification of microsatellites on sex chromosomes [65,186,187,188,189,190,191], some snake species, mainly colubrid snakes, have the highest density of microsatellites and general repeats in their genomes [185,192]. It has previously been documented that the major amniote taxa, including mammals and reptiles, exhibit remarkable variation in microsatellites [28,193,194,195]. High levels of microsatellite variability have been found in recent research among reptilian lineages, including snakes and other squamate reptiles [185]. In a number of investigations pertaining to the chromosome mapping of microsatellites in reptiles [196], the majority of microsatellites were shown to be distributed on sex chromosomes. It is hypothesized that sex chromosomes might have developed their specific appearance in each lineage because of rearranging after amplified microsatellite repeat motifs were preserved in the sex chromosomes of a common ancestor [197].

Peculiar evolutionary characteristics and the specialized biology of snakes have led scientists to consider them intriguing model systems for understanding how genomes change and how phenotypic-level evolution is linked to chromosomal diversity [198]. The number of repeat arrays of microsatellites in snake genomes is greater than that of any eukaryotic genomes discovered to date [185,197]. The estimated microsatellite content for colubrid snakes (e.g., *Coniophanes fissidens*) is 14% [185]. Even at the species level, snakes typically exhibit lineage-specific heterogeneity in the genomic abundance of identical microsatellite repeats as compared to other vertebrates (fish and mammals). Primitive snakes have the lowest density of these repeats, whereas sophisticated snakes have the highest, as shown by the repeatomic density analysis of microsatellites, resulting in variations ranging from 10.9 to 16.6 times. The enormous diversity of these repeats previously seen in fish genomes has been surpassed by the extreme heterogeneity of microsatellite genomic contents in snakes [192,199]. In contrast, microsatellite density variation is often lowest in mammalian and avian genomes (1.8-fold loci/Mbp and 2.8 bp/M). The prevalence of particular motifs (4-mer ATAG and 5-mer AATAG) with varying degrees of expansion in highly evolved snakes is an intriguing feature of microsatellite evolution in squamate genomes. How highly abundant these microsatellite motifs became in advanced snake genomes is still unknown. According to earlier research [185,197], excessive amounts of microsatellite genome expansion can result from a particular mechanism called “microsatellite seeding”. The substantial genomic prevalence of CR1-L3 long interspersed nuclear elements (LINEs) in areas close to microsatellites suggests that the growth of these transposons drives the microsatellite seeding process in colubroid snakes. Additionally, the vicinity of highly duplicated venom genes in snakes is characterized by enriched tandem repeat seeding [198,200,201]. In contrast, the house (*Hemidactylus frenatus*) and flat-tailed house (*H. platyurus*) gecko lizards did not exhibit large collections of microsatellite repeat patterns [202].

There are multiple research cases where the use of microsatellites has succeeded in assessing and preserving reptile species and populations. For example, microsatellite-based analysis using 22 marker loci was implemented for evaluating the genetic variability and population structure in two of Thailand’s most endangered species, the Siamese (*Crocodylus siamensis*) and saltwater (*C. porosus*) crocodiles [31]. A previous genetic bottleneck and a significant degree of genetic diversity were proposed by this study. For three individual crocodiles, microsatellite markers provided evidence of introgression, indicating that hybridization between *C. siamensis* and *C. porosus* may have taken place. Consequently, long-term conservation management depends on the identification of genetically hybrid and non-hybrid individuals. In addition, a unique technique that can make up for the shortcomings of each approach was developed by combining mtDNA and nuclear genetic data, including microsatellite genotyping and species-diagnostic SNP markers [203]. This approach makes it possible to prioritize conservation before releasing the species into the wild, guaranteeing long-term genetic integrity for management and reintroduction initiatives. “Ground truth” data on the relationship between the genotype and phenotypic variation in Siamese crocodiles in captive populations is crucial for future conservation efforts. In recent years, a call has been put out to redefine the importance of genetic admixture analysis for species conservation. In other words, robust protocols designed to identify introgression and hybridization are needed with all haste. Here, the genetic approach adopted by [31] established that combining information from the genotype (including microsatellites) and phenotype can assist in ensuring the long-term survival of the Siamese crocodiles through reintroduction programs plus in situ and ex situ management. Through this, sustainable genetic diversity can be maintained [31].

Populations of water monitors (*Varanus salvator macromaculatus*), huge lizards found in Thailand’s wetlands, appear to be decreasing as a result of habitat fragmentation brought on by urbanization. Based on microsatellite genotyping, genetic diversity at the population level was substantial. A captive study population was well established, according to genetic monitoring results; however, a possible transfer of water monitor groups in the future requires a comparison of allelic profiles between populations [204].

### 4.10. Amphibians

In vertebrates with Y or W sex chromosomes, microsatellite repeat motifs are frequently amplified [65]. In order to fully identify the sex chromosomes in rice field frogs (*Hoplobatrachus rugulosus*), Panthum et al. [205] used fluorescence in situ hybridization (FISH) mapping to discover 19 microsatellite repeat motifs and telomeric repeats. Using FISH, the chromosomal sites of 19 microsatellite repeat motifs and telomeric (TTAGGG)*_n_* sequences were identified. The findings of the FISH study showed that seven chromosomal pairs had interstitial signals, and hybridization signals showed that all chromosomes had TTAGGG repeats at their telomeric ends. All males and females exhibited hybridization signals for the microsatellite repeat motifs of (AGAT)_8_ in the subterminal region of the short arm of chromosome 1. The remaining 18 microsatellite repeat motifs did not provide any signals, supporting a non-genetic sex determination system in this species [205].

As with many amphibian species, the endemic Caucasian parsley frog (*Pelodytes caucasicus*) is experiencing a decline in population. In its four Turkish populations, genetic diversity and structure were estimated using microsatellite markers [206]. Because of rapid genetic drift, the populations exhibited moderate genetic differentiation despite their limited geographic distribution. In spite of a high inbreeding coefficient and little genetic variability, no indication of a genetic bottleneck was discovered. According to Papežík et al. [61], water frogs of the genus *Pelophylax* are among the most commonly translocated species outside of their natural range in Europe and have recently been observed on Malta’s Gozo island. To ascertain their population genetic structure and the anticipated number of source populations, a collection of microsatellite markers was analyzed. The lack of a population genetic structure and low genetic variability found by microsatellite research indicate that Gozo water frogs have a single source population. Using microsatellite loci for wood frogs, Winters et al. [207] evaluated population organization, gene flow, and genetic diversity within and between natural and artificial pools in a Pennsylvania state park. By maintaining comparable levels of genetic variation to natural pools, two thoughtfully constructed pools helped to sustain the local amphibian population. However, one badly designed pool served as a population sink and was genetically unique.

When it is difficult to distinguish between amphibian species, expressed sequence-tag (EST) SSRs that amplify across divergent lineages can be perfect. With a 67.67% interspecies transferability rate, cross-amplifying EST-SSRs derived from the transcriptomes of five endemic *Hynobius* salamander species in Taiwan were reported by Chen et al. [208]. Twenty polymorphic EST-SSRs with a high interspecies *PIC* (0.63) were utilized to evaluate interspecies genetic diversity and find individual markers displaying cross-species polymorphism. Notable between-cluster genetic divergence was revealed by pairwise *F*_ST_ values (>0.15). Other techniques have also confirmed that this set successfully categorized the individuals under study into five different clusters and is appropriate for long-term population genetic composition monitoring.

### 4.11. Fish

Microsatellite markers have found very wide application in fish genetics. To give a few recent examples, in a study by Hou et al. [209], the genetic variation and population structure of the Chinese longsnout catfish (*Leiocassis longirostris*) were assessed using 15 highly variable microsatellite DNA loci. This freshwater species is economically significant, despite a sharp loss in its wild resources. Catfish populations were shown to have substantial levels of genetic variety, but neither a systematic regional pattern of variation nor considerable genetic differentiation was found. Therefore, for within-river breeding initiatives and stock augmentation to restore the wild population, the source of the broodstock is not crucial. From the genome of the yellowfin seabream (*Acanthopagrus latus*), a marine fish of commercial significance in China and Southeast Asia, almost 319,000 SSRs were identified and described by Peng et al. [210]. These findings establish the basis for the molecular MAS and genetic information evaluation of *A. latus*, as well as cross-species microsatellite transferability in *A. schlegelii*. Six populations of the South Korean endemic *Microphysogobio longidorsalis* were analyzed using 19 microsatellite loci [211], which revealed a low level of genetic divergence and diversity among the populations. Thus, conservation measures are needed to keep *M. longidorsalis* from experiencing inbreeding depression. Because of catastrophic changes to the Aral Sea system, two local fishes, *Luciobarbus conocephalus* and *L. brachycephalus*, are under immediate danger of going extinct. The development of 15 new polymorphic microsatellite loci [212] has been effective and may be useful for population genetics, conservation, and other relevant studies of these species.

To foster population monitoring, ten new microsatellite markers from the Siamese fighting fish (*Betta splendens*), a popular ornamental fish and a novel model species, were isolated and characterized, and their applicability to related species, such as *B. smaragdina* and *B. imbellis*, was examined [213]. These microsatellite markers could be employed as a tool to study hatchery breeding strategies, genetic diversity, and population structure. The transferability of these loci was also confirmed in a study tackling genetic and environmental factors for conservation efforts in 17 *B. siamorientalis* populations [214]. These populations had a high level of genetic diversity without inbreeding or outbreeding. Further, over 810,000 microsatellite loci, making up 6.57% of the genome of *B. splendens*, were annotated [215]. These sequences were proposed as potential cross-species amplification markers and could help with MAS, population structure exploration, and genetic diversity evaluation.

As demonstrated by Suntronpong et al. [216], most chromosomes of the Asian swamp eel (*Monopterus albus*) had scattered mappings of 8 of the 19 microsatellite repeat motifs. This indicates that many microsatellite repeat motifs have been independently amplified in the *M. albus* genome. Remarkably, the microsatellite repeat motif signals’ dispersion was co-localized to the *M. albus* chromosomes along with *Rex* retroelements. This implies that during the evolution of the *M. albus* genome, microsatellite repeat motifs and *Rex* retroelements have both co-amplified. It was established [217] that the microsatellite repeat motif distribution in the jade perch (*Scortum barcoo*) was the largest in chromosome 19 (SBA19). Microsatellites were found in high quantity on SBA19 close to male-linked region 2. In several vertebrates, chromosomics, genome informatics, and molecular cytogenetics have shown a similar distribution of microsatellite repeats on sex chromosomes, indicating a potential role for these repeats in sex chromosome differentiation and evolution [190,218]. As followed from the overall analysis, SBA19 is the putative Y sex chromosome in this fish [217].

In the bighead catfish (*Clarias macrocephalus*), the North African catfish (*C. gariepinus*), and their sterile hybrids, microsatellite repeat motifs (CA)*_n_* accumulate differently [219]. This could indicate that the failure of homoeologous chromosome pairing brought on by genome-wide sequence divergence between the parental species is the cause of the disruption of spermatogenesis at the pachytene stage of male hybrids. In contrast, according to Lisachov et al. [220], microsatellite repeat motif differential accumulation may not be a factor in hybrid sterility, which requires further investigation. Because the North African catfish is a significant species in aquaculture, which is crucial for ensuring food and nutrition security, it is introduced in other countries. Three populations captured for breeding in Thailand were subjected to microsatellite genotyping in order to examine their genetic diversity and the underlying cause of potential inbreeding depression [221]. Intriguingly, each population had incredibly low inbreeding coefficients, and the three populations showed clear genetic diversity, suggesting that their genetic backgrounds differ significantly. This demonstrates that the reduced productivity of the North African catfish in Thailand may be due to outbreeding depression caused by genetic mixing among currently captured populations of different origins [222,223,224,225,226].

Collectively, the considered applications of microsatellite markers are instrumental molecular tools for studying genomic variability and other issues in vertebrate species/populations [227,228,229,230,231,232,233,234] (as summarized in Table 2).

## 5. Conclusions

Microsatellites are a distinct class of molecular genetic markers [235,236,237,238] that are widely used to address a number of issues pertaining to the scientific support of breeding work, specifically that of animal breed certification, the degree of genetic differentiation of populations, and the degree of consolidation of newly created animal groups [239,240,241]. A thorough analysis of genetic resources, the management of breeding conditions, and the creation of strategies for maintaining the gene pool in a confined population is an additional challenge in animal genetics and breeding [242,243,244,245]. Combining/comparing SNP and microsatellite analysis [246,247,248,249] can be crucial when constructing phylogenetic trees in evolutionary investigation [69,250], studying the patterns of genetic structure dynamics [251,252], and monitoring gene pool preservation [253,254,255,256,257,258]. Insight into the association of different alleles with economically important traits is also a necessary component for conducting effective marker-assisted breeding work [36,38,259]. In this regard, the necessity for molecular scientists to always maintain broad horizons, avoid excessive specialization, and consider areas in which their work may be applied in a different disciplines is paramount. Microsatellites have not been replaced by SNPs for such analyses; rather, the two continue to work in tandem.

## Figures and Tables

**Table 1:** Types of microsatellite-specific computer programs used for the population genetic analysis.

Name	Function	Reference
MICRO-CHECKER	Investigates microsatellite data, calculates simple summary statistics, and shows the potential for mistyped and null alleles	[93]
Microsatellite Toolkit	A practical Excel microsatellite data handling tool that offers summary statistics (the number of alleles observed and expected heterozygosity and allele frequencies) and verifies the dataset for errors	[94]
Power Marker	A feature-rich Windows application that offers a variety of summary statistics, genetic distances and bootstrapped phylogenetic trees for microsatellites, SNPs, and other biallelic data	[95]
Msvar	Uses microsatellite frequencies to identify a previous population expansion or decline	[96]

**Table 2:** Summary of Section 4 indicating species covered and the benefits of microsatellite studies on them.

Type/Species	Subtype/Breed	Types of Study	Main Findings	References
**Humans/other primates**	Apes, baboons, macaques, and certain platyrrhine monkeys	Mostly telomere repeats	Weak conservation among monkey lineages; humans/monkeys have similar sequence lengths	[119,120,121,122,123,124,125,126,127,128,129,130,131,132,133,134,135,136,137]
**Cattle**	Short statured Nattukuttai	Bottleneck analysis	No population decline	[138]
	Gabrali	Genetic diversity, 12 loci	Substantial genetic diversity and does not face threats of inbreeding/bottlenecks	[46]
	Lebedyn	10 FAO-ISAG-recommended loci	Genetic equilibrium and propensity for inbreeding in some breeds	[139]
**Other Artiodactyla**	Chinese goral	Population genetics; 11 loci	Low genetic variation due to inbreeding and a small effective population size in captivity; management as evolutionarily significant units recommended	[141]
	Mouse-deer	Genotyping and demographic analysis	No historical bottleneck, a reduced effective population size, and inbreeding, raising extinction risk. Improved care boosted population growth	[142]
**Horses**	Korean native horse	Parentage verification	Early application of 16 STRs for pedigree control; foundation for the national ID system	[144]
	Thoroughbred, Jeju, Sumbawa, and Kazakh	Parentage testing, breed certification, diversity, and conservation	High heterozygosity in Thoroughbreds; low diversity in Sumbawa; admixture in Kazakh horses; ISAG STR panel validated	[145,146,148]
**Donkeys**	Korean domestic donkeys	Genetic diversity, breed identification, and conservation	Lower heterozygosity than horses; clear species distinction; 9 STR loci validated	[147]
	Mediterranean and Asian breeds	Genetic structure and conservation status	Moderate diversity; breed-specific structure (e.g., Pantesco); highlights importance of structured conservation programs	[149]
**Chickens**	Canarian population	Genetic variation	High variation and did not cluster with other Spanish breeds	[154,155]
	Nagoya breed	Breed discrimination	4 Nagoya breeds identical to the *ABR0417* reference sequence	[156]
	Ukrainian breeds	Genetic variation	Largest genetic differences found between Plymouth Rock White and Rhode Island Red and smallest between the Plymouth Rock White and Poltava Clay breeds	[38,157,158,159,160]
	Indigenous/Red Jungle fowl	Genetic diversity and population structure; 28 ISAG-FAO loci	High genetic variability; evidence of genetic introgression; selection pressures in fighting cocks; distinct clustering of Thai local breeds; importance of red junglefowl gene pool for reintroduction	[60,62,79,153,161,162,163,164]
	Lao Pa Koi	Genetic admixture and diversity; 28 loci	Shared partial gene pool with red junglefowl; high genetic diversity	[163]
	Lueng Hang Khao	Genetic admixture, diversity; 28 loci	Hitchhiking selection, indicating directional selection in fighting cocks	[164]
	Pradu Hang Dam	Genetic admixture, diversity; 28 loci	Partial gene pool overlap, suggesting that Samae Dam may be variety of Pradu Hang Dam	[162]
	Chinese black-boned chicken	Population structure	Originated from a native Chinese chicken with introgression from the red junglefowl and other domestic breeds	[62]
**Other birds**	Hume’s pheasant	Genetic diversity and population structure	High genetic diversity in wild populations but low differentiation and inbreeding in Thai captive flocks; findings and conservation efforts	[166,167,168]
	Asian woolly-necked storks	Genetic diversity, population structure, demographic history, and captive and reintroduced populations	Captive breeding caused inbreeding and a small effective population in one population, while another showed signs of a recent bottleneck; in oriental storks, prolonged captive propagation stabilized genetic diversity, highlighting the need for genetic assessments in reintroduction efforts	[169]
	California condor	Various factors/phenomena and variation	Established parentage, facultative parthenogenesis, and linkage map	[44,171,172,173]
**Dogs**	Labrador, German Shepherd etc.	Parentage testing	No signs of inbreeding, sufficient for establishing dog parentage	[175]
	Bangkaew and Thai Ridgeback	Genotyping and genetic diversity	Bangkaew dogs exhibit significant genetic variation with low inbreeding risk; Thai Ridgebacks maintain high genetic diversity with no bottlenecks	[176,177]
	American Kennel Club breeds	Differentiation within breeds	Breed-to-breed genetic relatedness less clear-cut; autosomal microsatellite set proved helpful in characterizing genetic variation within breeds	[174]
**Cats**	*Felis*, *Panthera* and *Prionailurus*	Genotyping and comparative analysis	Domestic cats have a higher microsatellite frequency than wild cats, providing extensive genetic resources	[178,179]
	Siamese and Korat	Genetic diversity and population structure	Moderate genetic diversity and high inbreeding; broader studies of Thai cat breeds reveal high genetic diversity and distinct gene pool patterns	[180]
**Elephants**	African and Asian	Genotyping and population genetics	Cryptic speciation in Asian elephants; in captive Thai elephants, genetic diversity varied across populations and declined over 50 generations	[30,45,67,182,183,184]
	Woolly mammoths	Demographic history until extinction	Reduction in genetic variation before extinction 4000 years ago	[59]
**Reptiles**	General studies	Microsatellite identification	Remarkably abundant in squamate reptile genomes, majority of microsatellites distributed on sex chromosomes; lower abundance in geckos	[28,185]
	Snakes	Abundance, distribution, and evolution	Particularly colubrids, highest density of microsatellites among vertebrates; most enriched on sex chromosomes; microsatellite expansion driven by transposable elements that are linked to venom gene duplication	[28,65,185,186,187,188,189,190,191,192,193,194,195,196,197,198,199,200,201]
	Siamese/saltwater crocodiles	Endangered species genotyping, genetic variability, and population structure	Evidence of past genetic bottlenecks and hybridization. Identified hybrids for long-term conservation management to enhance conservation strategies	[31]
	Water monitors	Genetic diversity and population structure	Substantial genetic diversity despite habitat fragmentation due to urbanization; well-established captive population identified through genetic monitoring; future relocation efforts require allelic profile comparisons	[204]
**Amphibians**	Rice field frogs	FISH mapping of repeat motifs and telomeric sequences	Identified 19 microsatellite repeat motifs and telomeric sequences; the absence of sex-specific signals suggests a non-genetic sex determination system	[205]
	Caucasian parsley frog	Population studies	Population decline, rapid genetic drift, and moderate genetic differentiation	[206]
**Fish**	Chinese longsnout catfish	Genetic variation and population structure	Substantial genetic variety; neither a systematic regional pattern of variation nor considerable genetic differentiation	[209]
	Yellowfin seabream	Population studies; 19 loci	Low level of genetic divergence and diversity conservation measures needed	[210]
	Siamese fighting fish and related species	Development and characterization of markers	Ten new markers identified and characterized; they are used for hatchery breeding strategies, genetic diversity assessments, population monitoring, marker-assisted selection (MAS), and conservation efforts	[213,214,215]
	Asian swamp eel, Jade perch	Chromosomal mapping and distribution analysis	Eight microsatellite repeat motifs scattered across most chromosomes that are co-localized with retroelements, suggesting co-amplification during evolution; in Perch highly concentrated on chromosome 19, the putative Y chromosome	[216,217]
	Bighead catfish	Hybrid sterility and genetic diversity	(CA)n microsatellite-differential accumulation, possibly disrupting spermatogenesis. Low inbreeding but high genetic diversity, suggesting potential outbreeding depression	[219,220,221]

## Abbreviations

The following abbreviations are used in this manuscript:

ACO	ant colony optimization
dNTP	deoxynucleoside triphosphate
EST	expressed sequence-tag
FAO	Food and Agriculture Organization of the United Nations
*F*_IS_	inbreeding coefficient of individuals in a subpopulation
FISH	fluorescence in situ hybridization
*F*_IT_	inbreeding coefficient of individuals in the population as a whole
*F*_ST_	inbreeding coefficient of the subpopulation relative to the entire population
GC-content	guanine-cytosine content
*H_e_*	expected heterozygosity
*H**_o_*	observed heterozygosity
HRM	high-resolution melting
ISAG	International Society of Animal Genetics
ISSR	inter simple sequence repeat
LINEs	long interspersed nuclear elements
MAS	marker-assisted selection
mtDNA	mitochondrial DNA
PCR	polymerase chain reaction
*PIC*	polymorphic information content
POT1	protection of telomeres 1
QTLs	quantitative trait loci
RAPD	random amplified polymorphic DNA
RFLP	restriction fragment length polymorphism
SNP	single nucleotide polymorphism
SSR	simple sequence repeat
STRs	short tandem repeats
TRF1	telomeric repeat binding factor 1
TRF2	telomeric repeat binding factor 2
